# Supplementary material for: The structural basis for RNA selectivity by the IMP family of RNA-binding proteins
Source: Nat Commun. 2019 Sep 30;10:4440. doi: 10.1038/s41467-019-12193-7 (PMC6768852; doi:10.1038/s41467-019-12193-7)
Supplement: Supplementary file 3 — Reporting Summary [file 41467_2019_12193_MOESM3_ESM.pdf]

nature research

Corresponding author(s): Robert H. Singer, Carolina Eliscovich

Last updated by author(s): Apr 29, 2019

Reporting Summary

Nature Research wishes to improve the reproducibility of the work that we publish. This form provides structure for consistency and transparency in reporting. For further information on Nature Research policies, see Authors & Referees and the Editorial Policy Checklist .

Statistics

For all statistical analyses, confirm that the following items are present in the figure legend, table legend, main text, or Methods section.

n/a	Confirmed	
☒	☐	The exact sample size (*n*) for each experimental group/condition, given as a discrete number and unit of measurement
☐	☒	A statement on whether measurements were taken from distinct samples or whether the same sample was measured repeatedly
☐	☒	The statistical test(s) used AND whether they are one- or two-sided<br>*Only common tests should be described solely by name; describe more complex techniques in the Methods section.*
☒	☐	A description of all covariates tested
☐	☒	A description of any assumptions or corrections, such as tests of normality and adjustment for multiple comparisons
☐	☒	A full description of the statistical parameters including central tendency (e.g. means) or other basic estimates (e.g. regression coefficient) AND variation (e.g. standard deviation) or associated estimates of uncertainty (e.g. confidence intervals)
☒	☐	For null hypothesis testing, the test statistic (e.g. *F*, *t*, *r*) with confidence intervals, effect sizes, degrees of freedom and *P* value noted<br>*Give P values as exact values whenever suitable.*
☒	☐	For Bayesian analysis, information on the choice of priors and Markov chain Monte Carlo settings
☒	☐	For hierarchical and complex designs, identification of the appropriate level for tests and full reporting of outcomes
☒	☐	Estimates of effect sizes (e.g. Cohen's *d*, Pearson's *r*), indicating how they were calculated

Our web collection on statistics for biologists contains articles on many of the points above.

Software and code

Policy information about availability of computer code

Data collection	NMR Data was acquired using manufacturer standard Bruker and Varian software and pulse sequences. EMSAs were scanned on Typhoon 9400 (GE Healthcare), using manufacturer standard software.
Data analysis	NMR data was processed with NMR Pipe and analyzed with CCPN, PINE and NMRFAM-Sparky. EMSAs were analyzed with FIJI/ImageJ using intensity measurements. Graphs and fits were generated in Prism 7 (Graph Pad). IPA Knowledge Base 9 (Ingenuity Systems) was used to determine diseases and functional category enrichment. RNA seq TPMs were generated with Kallisto (Patcher Lab).

For manuscripts utilizing custom algorithms or software that are central to the research but not yet described in published literature, software must be made available to editors/reviewers. We strongly encourage code deposition in a community repository (e.g. GitHub). See the Nature Research guidelines for submitting code & software for further information.

Data

Policy information about availability of data

All manuscripts must include a data availability statement. This statement should provide the following information, where applicable:

- Accession codes, unique identifiers, or web links for publicly available datasets
- A list of figures that have associated raw data
- A description of any restrictions on data availability

Crystal structure was deposited to the PDB under code 6ROL (Fig 1, Sup Fig. 4), NMR data (Fig 3, Sup Fig. 3) was deposited to the BMRB under accession number 27934. The source data underlying Figs. 2a, 2f and Supplementary Fig 7 are provided as a Source Data file. All other relevant data are available from the authors upon request.

Field-specific reporting

Please select the one below that is the best fit for your research. If you are not sure, read the appropriate sections before making your selection.

- [x] Life sciences
- [ ] Behavioural & social sciences
- [ ] Ecological, evolutionary & environmental sciences

For a reference copy of the document with all sections, see nature.com/documents/nr-reporting-summary-flat.pdf

Life sciences study design

All studies must disclose on these points even when the disclosure is negative.

Sample size	Based on previously published literature.
Data exclusions	EMSAs were RNA was not visible or under-labeled were repeated with freshly labeled RNA. For mutations that were unable to bind at 1uM protein, Kd values were either repeated with higher protein concentration or not calculated, instead being reported as no significant binding.
Replication	EMSAs were performed at least 3 times, representative gels and quantifications are shown. Reported Kds are representative of all samples but for clarity only data points and fit for a single gel are shown.
Randomization	Does not apply
Blinding	Does not apply

Reporting for specific materials, systems and methods

We require information from authors about some types of materials, experimental systems and methods used in many studies. Here, indicate whether each material, system or method listed is relevant to your study. If you are not sure if a list item applies to your research, read the appropriate section before selecting a response.

Materials & experimental systems

n/a	Involved in the study	
x		Antibodies
x		Eukaryotic cell lines
x		Palaeontology
x		Animals and other organisms
x		Human research participants
x		Clinical data

Methods

n/a	Involved in the study	
x		ChIP-seq
x		Flow cytometry
x		MRI-based neuroimaging
