# Supplementary material for: The structural basis for RNA selectivity by the IMP family of RNA-binding proteins
Source: Nat Commun. 2019 Sep 30;10:4440. doi: 10.1038/s41467-019-12193-7 (PMC6768852; doi:10.1038/s41467-019-12193-7)
Supplement: Supplementary file 4 — Description of Additional Supplementary Files [file 41467_2019_12193_MOESM4_ESM.docx]

**Description of Additional Supplementary Files**

File Name: Supplementary Data 1
Description: Sequences conserved between humans and mice for IMP2 binding show enrichment for metabolic genes related to diabetes

File Name: Supplementary Data 2
Description: RNA oligonucleotides used in this study
