# Supplementary material for: The structural basis for RNA selectivity by the IMP family of RNA-binding proteins
Source: Nat Commun. 2019 Sep 30;10:4440. doi: 10.1038/s41467-019-12193-7 (PMC6768852; doi:10.1038/s41467-019-12193-7)
Supplement: Supplementary file 6 — Source Data [file 41467_2019_12193_MOESM6_ESM.zip › Figure-2-SELEX Sequence Source Data.docx]

**IMP2KH34 Round 7 sequences**

GTCGCGTTCGGACAAGAGAATATTAAGGG

GTCGCGTTCGGACAAGAGAATATTAAGGG

GAATACAGCGAACCCTTAGGAGACACTAT

CCTCCGCTAGTCAAATCAGCCNCGTTCCTT

TGTCACGCGTTTCAGCTCTCCCTTTGGGT

TGGACAGTCAGGGCGAACGTACTGGGGAC

CCTCCGCTAGTCAAATCAGCCTGTTCCTT

CCTCCGCTAGTCAAATCAGCCTGTTCCTT

ATTACAGCGTCCGCCAGTTGACTGAATAG

GGGTCAAGTTCGCCGTAGGATATAATGTT

GGGCCAGCGAACCCTTCGGAAATACCTGT

TTAATGCAACAAGAATCTACCGGTCCCGT

GGATTCAGTGAACCCAGTTGGAAATCTT

GGAGTCAGCGAACCCCTTGGACCCTTATG

GGAGTAAGCGAACCCTTAGGTCTAGTTGT

CACTGCGAATTCAGCGAACCCTCAGGTAT

CACTGCGAATTCAGCGAACCCTCAGGTAT

CACTGCGAATTCAGCGAACCCTCAGGTAT

GTAGTCACCTCACCCTCTGGAATTTGCTT

AATATTGTATGAACACCCCGAATTCCTTC

GAGTTCAGCTCACCCTTTGGTTATTTGCT

AAATCTTCCTATCTGGTCAACACCCGTTGA

AAATCTTCCTATCTGGTCAACACCCGTTGA

GAATACAGCGAACCCTTAGGAGACACTAT

CACTGCGAATTCAGCGAACCCTCAGGTAT

AGGTTTAATGCGTCNAGATTGAATCCTCC

ATTACCCCTTGGGTCATCCGAGGGCGAAC

GCTTCAAGTTCTGCCTTTGGTAATAGTCT

GGGCCAGCGAACCCTTCGGAAATACCTGT

CTGTGTATACAGCGAACCCTTAGGATGAT

GCTTCAAGTTCTGCCTTTGGTAATAGTCT

GTACTCAGCGAACCCCTTGGAATGCTTCT

GAATACAGCGAACCCTTAGGAGACACTAT

TGCGATCCAGCGAACCCATGGGATGTTTC

GAGTTCAGCTCACCCTTTGGTTATTTGCT

GTAGTCACCTCACCCTCTGGAATTTGCTT

GCTTCAAGTTCTGCCTTTGGTAATAGTCT

GAGTTCAGCTCACCCTTTGGTTATTTGCT

TGCGAACCAGCTTACCCCTTGGATATCCT

TCCTAGGCTGGCCGTCTCTAAAGTGCTCC

ACCACAGCGGGTCAGTTCTCCCTTAGGAT

GGAAACAGTGAACCCCGTTGGAAGAAAAT

CGGAGAAATGCTCTTGCAACAAGTCATGG

AGGTTTAATGCGTCAAGATTGAATCCTCC

CGCTGCGTATACAGTGAACCCTTTGGAGT

CGCTGCGTATACAGTGAACCCTTTGGAGT

**Round 7 – 46 sequences**

| GG ELEMENTS | CA ELEMENTS |
| --- | --- |
| AGGT – 7 – 15.2% | TCA – 23 – 50% |
| TGGA – 10 – 21.7% | CCA – 6 – 13% |
| AGGA – 6 – 13% | ACA – 13 – 28% |
| TGGT – 8 – 17.4% | GCA – 2 – 4.3% |

**IMP2KH34 Round 8 sequences**

GTAAACAGCGAACCCCTAGGAACAGATCTT

GTAGTCACCTCACCCTCTGGAATTTGCTTC

GTAGTCACCTCACCCTCTGGAATTTGCTTC

GGAGTAAGCGAACCCTTAGGTCTAGTTGTC

GTAAACAGCGAACCCCTAGGAACAGATCTT

GGAGTAAGCGAACCCTTAGGTCTAGTTGTC

GAGTACAGCGAACCCCTNCGGTCAACGATTC

GTAGACACCTTACCCTTCGGAGAACTTTTC

GTAGTCACCTCACCCTCTGGAATTTGCTTC

GTAGTCACCTCACCCTCTGGAATTTGCTTC

CACTGCGAATTCAGCGAACCCTCAGGTATC

GTAGTCACCTCACCCTCTGGAATTTGCTTC

GGAGTAAGCGAACCCTTAGGTCTAGTTGTC

GTAGTCAGTGAACCCTCTGGAAGAATTCTC

GAATTCAGCGTCTCCCACTGGAACTTTCTC

GAGAATTGCGCTTAAAGTTCGCCCTTGGG

GATCCAGTGAACCCTTTGGACTTATTCGAT

GTAGTCACCTCACCCTCTGGAATTTGCTTC

GGGACAAGCTCTCTCCTAGGTTAATTGGTC

GGGACAAGCTCTCTCCTAGGTTAATTGGTC

GGATTCAGTGAACCCAGTTGGAAATCTTC

GGATTCAGTGAACCCTTTTGGAAAACCTTC

CACTGCGAATTCAGCGAACCCTCAGGTATC

ACCACAGCGGGTCAGTTCTCCCTTAGGATC

CTGTGTATACAGCGAACCCTTAGGATGATC

GGAGTAAGCGAACCCTTAGGTCTAGTTGTC

ACCACAGCGGGTCAGTTCTCCCTTAGGATC

CTGTGTATACNAGCGAACCCTTAGGATGATC

TGCGATTCAGCTTACCCTTAGGTCTCTACT

CTGTGTATACAGCGAACCCTTAGGATGATC

CACTGCGAATTCAGCGAACCCTCAGGTATC

GGATTCAGTGAACCCAGTTGGAAATCTTC

GTAGTCACCTCACCCTCTGGAATTTGCTTC

TCCCAGCTACTTGTGACTTAGCTCTTCACA

CTGTGTATACAGCGAACCCTTAGGATGATC

TGCGATTCAGCTTACCCTTAGGTCTCTACT

GGGTTAGCGAACCCTTTGGAAAATATTGGC

GGAGTAAGCGAACCCTTAGGTCTAGTTGTC

CTGTGTAGACAGCGAACCCTTAGGATGATC

TGCGGGCTCGGCGCTGCCCTTTGGTATCTC

GTAGTCACCTCACCCTCTGGAATTTGCTTC

CTGTGTATACAGCGAACCCTTAGGATGATC

GGAGTAAGCGAACCCTTAGGTCTAGTTGTC

**Round 8 – 43 sequences**

| GG ELEMENTS | CA ELEMENTS |
| --- | --- |
| AGGT – 13 – 30.2% | TCA – 21 – 48.8% |
| TGGA – 15 – 34.9% | CCA – 4 – 9.3% |
| AGGA – 10 – 23.2% | ACA – 14 – 32.5% |
| TGGT – 3 – 7.0% | GCA – 0 – 0% |

**IMP2KH34 Round 9 sequences**

GGCCTCAGCGAACCCTTTGGAACACTTCTC

CTGTGTATACAGCGAACCCTTAGGATGATCGCA

CTGCGGATCCAGCAAACCCCTTGGGTAGTC

GGATTCAGTGAACCCAGTTGGAAATCTTC

GGCCGCGAATTCGCCCTTGGGCGAATTCGTTTAAACCTGCAGGACTAGTCCCTTAGTGAGGGTAA

GGATTCAGTGAACCCAGTTGGAAATCTTC

TGGCAGATTCAGCTCTCCCTTAGGTACATC

GGAGTAAGCGAACCCTTAGGTCTAGTTGTC

AGAATTCAGCGAACCCAAAGAGACTTGCCC

GTGAGTCAAGGGCGAATTCGTTTAAACCTGCAG

GGATTCAGTGAACCCAGTTGGAAATCTTC

GTACTCAACTCTCCCATTGGGCTTGTGTTC

TACGTTGACAACTCACCCTTTGGGAATTTC

TACGTTTCAAGCTCGCTCTTGGGGATATTC

GGAGTAAGCGAACCCTTAGGTCTAGTTGTC

GGATTCAGCGAACCCTTGGGAGACTTCTTC

GGATTCAGTGAACCCAGTTGGAAATCTTC

GGCCACAGCGAACCCATTGGAACCTTCTC

GGAGTAAGCGAACCCTTAGGTCTAGTTGTC

GGATTCAGTGAACCCTTTTGGAAATCTTCC

GGAGTAAGCGAACCCTTAGGTCTAGTTGTC

GGATTCAGTGAACCCAGTTGGAAATCTTC

GGATTCAGTGAACCCAGTTGGAAATCTTC

GTAATCACCTCACCCTTCGGATCTAATCTC

GAATTCAGCGAACCCATTGGAACCTTCTC

GGATTCAGTGAACCCAGTTGGAAATCTTC

TGCGAACCAGCTTACCCCTTGGATATCCTC

GTACACAGCGAACCCATTGGAACCTTCTC

CACTGCGAATTCAGCGAACCCTCAGGTATC

GAATTCAGCGAACCCTTGGGAGACTTCTTC

GGATTCAGTGAACCCAGTTGGAAATCTTC

GGATTCAGTGAACCCAGTTGGAAATCTTC

GCCCCAAGTTCTGCCTTTGGATATGTTGTC

GGAGTAAGCGAACCCTTAGGTCTAGTTGTC

GGATTCAGTGAACCCAGTTGGAAATCTTC

GGGACAAGCATTGCCCTTTGGTTTAGAATC

CACTGCGAATTCAGCGAACCCTCAGGTATC

GGAGTAAGCGAACCCTTAGGTCTAGTTGTC

GTAGTCACCTCACCCTCTGGAATTTGCTTC

GGAGTAAGCGAACCCTTAGGTCTAGTTGTC

GGATTCAGTGAACCCAGTTGGAAATCTTC

GGATTCAGTGAACCCAGTTGGAAATCTTC

GGATTCAGTGAACCCAGTTGGAAATCTTC

GGATTCAGTGAACCCAGTTGGAAATCTTC

CTGTGTATACAGCGAACCCTTAGGATGATC

GGAGTAAGCGAACCCTTAGGTCTAGTTGTC

GGAGTAAGCGAACCCTTAGGTCTAGTTGTC

GGATTCAGTGAACCCAGTTGGAAATCTTC

GGAGTAAGCGAACCCTTAGGTCTAGTTGTC

GGATTCAGTGAACCCAGTTGGAAATCTTC

GGAGTAAGCGAACCCTTAGGTCTAGTTGTC

GGAGTAAGCGAACCCTTAGGTCTAGTTGTC

TGCGAACCAGCTTACCCCTTGGATATCCTC

GGAGTAAGCGAACCCTTAGGTCTAGTTGTC

GGAGTAAGCGAACCCTTAGGTCTAGTTGTC

GGCCGCGAATTCGCCCTTCTATCGGAATAAGGGCGAATTCGTTTAAACCTGCAGGA

GGATTCAGTGAACCCAGTTGGAAATCTTC

**Round 9 – 57 sequences**

| GG ELEMENTS | CA ELEMENTS |
| --- | --- |
| AGGT – 17 – 29.8% | TCA – 32 – 56.1% |
| TGGA – 27 – 47.3% | CCA – 7 – 13.2% |
| AGGA – 4 – 7.0% | ACA – 5 – 8.8% |
| TGGT – 1 – 1.8% | GCA – 4 – 7% |
