# Supplementary material for: The structural basis for RNA selectivity by the IMP family of RNA-binding proteins
Source: Nat Commun. 2019 Sep 30;10:4440. doi: 10.1038/s41467-019-12193-7 (PMC6768852; doi:10.1038/s41467-019-12193-7)
Supplement: Supplementary file 2 — Peer Review [file 41467_2019_12193_MOESM2_ESM.pdf]

Reviewers' comments:

Reviewer #1 (Remarks to the Author):

The manuscript by Biswas et al describes the structure of the IMP2 KH3-KH4 construct and investigates its RNA binding specificity using SELEX. The authors observed that the optimal RNA sequence bound by IMP2 differs slightly from the one bound by the homologous protein ZBP1. Using mutagenesis of the protein and the RNA, they provide a rationale for this difference.

Overall, I do have some concerns that are listed below:

1- Figure 1C shows an EMSA of ZBP1 and IMP2 with the C28 Zipcode RNA. From these data, I do not understand how the authors can derive a Kd of 161nM for the IMP2-C28 interaction since the interaction did not reach saturation at 2uM protein. I believe that the only conclusion here is that IMP2 has a lower affinity than ZBP1 for this RNA.

2- It is not clear what is the RNA sequences described in Supplementary Figure 1 (RNA #1 and RNA #2). Also in relation to Figure 1a, it would be useful to add a sequence alignment of the RNAs derived from SELEX round 9 as a supplementary figure. The authors should explain or speculate on the fact that 26% of the sequences contain a UAA motif at the 5'end instead of a UCA.

3- Most of the manuscript's conclusions are based on comparing the Kd of ZBP1 and IMP2 to different RNAs using quantifications of EMSA experiments (Figures 2c,d,e, and 5a,c,d, Sup Figs 1b,d, 2b). Are these derived from a single EMSA experiment? There are no standard deviations and statistical analysis of the results. These should be added to demonstrate that the results presented are statistically significant.

4- The figure presenting the NMR data (Fig. 3) is quite small and it is difficult to appreciate the changes in chemical shifts. Although Supp Figure 3 shows a larger version of the spectrum, it is not overlaid with the RNA bound form. Figure 3 A and B should be enlarged (possibly in a Supplementary Figure) and show an overlay of all the spectra (as in the examples shown in Supp. Fig. 3c and d). It would also be informative to add a bar chart of the chemical shift changes (or peak intensity change for KH4) as a function of the amino acid sequence. Also, what is the affinity of IMP2 to these RNA sequences? It seems that the binding of KH3 to CCUCACC is in fast exchange and the binding of KH4 to UUUGGAAC is in intermediate exchange. This is not consistent with the described low nanomolar affinities obtained with EMSA. Finally, how does the NMR analysis of the affected residues correlates with previous NMR studies of ZBP1 KH3-KH4 with RNA?

5- The mutagenesis analysis done to provide a rationale for the differential RNA binding between ZBP1 and IMP2 is unclear to me:
a. The previous experiments, especially SELEX, have shown that IMP2 binds a slightly different sequence than ZBP1. However, in the mutagenesis experiments, the authors have mutated IMP2 and investigated the binding of these mutants to the Zipcode RNA sequence. It would have been more logical to mutate ZBP1 residues and investigate their binding to the IMP2 RNA binding sequence obtained by SELEX.
b. The mutations are based on a sequence alignment of ZBP1 and IMP2. However, the structures of ZBP1 KH3 and KH4 in complex with RNA have been solved and this manuscript describes NMR data that should provide a detailed description of the amino acids involved in the RNA binding (see point 4 above). Are the mutated residues involved in RNA binding either in the published structures, or in the NMR titration experiments?
c. Similarly, RNA mutations were done on the Zipcode RNA and therefore do not explain why the SELEX RNA sequence is preferentially bound by IMP2 and less so by ZBP1. It would have been more judicious to mutate the SELEX RNA and investigate the binding of ZBP1 to these mutants.

6- The genome wide search for IMP2 targets is highly speculative and only based on the identification of sequences that correspond to the SELEX sequence identified for IMP2. There is no experimental evidence that the identified sequences are real targets of IMP2. At least, the functional interaction of IMP2 with some of the mRNAs encoding for metabolic diseases such as Type II diabetes should be demonstrated experimentally.

Reviewer #2 (Remarks to the Author):

The paper by Biswas and colleagues report on the structure and RNA binding properties of the KH3KH4 di-domain of the protein IMP2, one of three members of the IMP family in human. The authors show the structure of the di-domains of IMP2 is very similar to the one of the better characterized IMP1 paralogue. They then characterize the binding sequence of the di-domain and find that, while the two core positions of the sequence recognized by KH3 and KH4 are the same in IMP2 and IMP1, the bases flanking this di-nucleotide are different. They also show that these difference are due to sequence differences in the variable loop of the KH domains and that an IMP2 chimera with an IMP1 variable loop recognize the IMP1 cognate sequence. They search for targets containing this sequence and find that the identified putative IMP2 targets relate to metabolism control and that the overlap between putative targets containing the IMP1 and the IMP2 sequences could be significantly smaller than previously reported.
The paper is well-written and clear and the data present are logically organized and of good quality. It has a number of points of interest, including the structure of the di-domain, the novel sequence specificity, the creation of a chimera as a strategy to define specificity and, importantly, the definition of IMP1 and IMP2 sets of targets which have only a small overlap, which is different from what previously reported. Below are a number of comments to be addressed prior to publication.
1) The authors use the newly defined bi-partite RNA target motif to identify a number of bound 3’UTRs. They should directly validate the specificity of a selection these interactions. One possibility would be to use RIP or equivalent methods.
2) The authors show that IMP2 sequence specificity is different than IMP1. The increase in affinity for IMP2, and decrease for IMP1, as we proceed in the SELEX selection for IMP2 is a good validation that strengthens their conclusions. However, my understanding is that in the discussion SELEX is implicitly equated to actual evolution - it should be clarified this is not the case.
3) The authors show that most of the targets that they attribute to IMP2 are linked to metabolic disorders. This I find very interesting and I think it would be worth discussing at the level of individual targets in the discussion.
4) The overlap between the targets of IMP1 and IMP2 is much smaller than the one observed in CLIP studies. This is important, as it would change significantly the way we think about the role and targets of these proteins. It should be discussed more in my opinion.
5) Changing the sequence specificity of a KH domain by the mutation of an individual amino acid has been previously shown to be possible by Nicastro et al., NSMB 2012. This should be mentioned appropriately when discussing mutational strategies in more general terms.
6) The authors test the relevance of the variable loop in IMP2. What they find may not be true for other KH domains, for example the ones showing very little sequence specificity in ‘position 1’ of the bound sequence. This should be considered in the discussion.

To the reviewers:

Both reviewers suggested that we validate the IMP2 binding consensus sequence prediction with an orthogonal method such as RIP. We agree that the correlation of the consensus sequence with RIP targets would help strengthen the results we found. However, it should be noted that the IMP2 consensus element search makes predictions for both human and mouse genomes, therefore, any human or mouse cell line used for experimental validation could be appropriate for RIP but would only contain a subset of our RNAs containing the consensus element.

To validate the IMP2 consensus sequence, we have queried the high confidence IMP2 targets (those conserved between human and mouse) in the following published data sets and plotted their enrichment relative to input/controls as a new supplementary figure 7:

For human cells:

- Queried recently published RIP seq from HEK239T cells and determined fold enrichment relative to input (data processed from Huang et al 2018, Nature Cell Biology).

For mouse cells:

- Queried published RIP seq from brown adipose tissue and determined fold enrichment relative to IgG control (data processed from Dai et al 2015, Cell Metabolism).

We observed that a highly significant portion ($p < 0.0001$) of the predicted IMP2 consensus sequence targets were enriched in these previously published RIP studies. We have now included these results in a new supplementary figure (Sup Fig. 7) and in the text, accordingly.

Point-by-point response to the reviewers:

Reviewer #1 (Remarks to the Author):

The manuscript by Biswas et al describes the structure of the IMP2 KH3-KH4 construct and investigates its RNA binding specificity using SELEX. The authors observed that the optimal RNA sequence bound by IMP2 differs slightly from the one bound by the homologous protein ZBP1. Using mutagenesis of the protein and the RNA, they provide a rationale for this difference.

Overall, I do have some concerns that are listed below:

1- Figure 1C shows an EMSA of ZBP1 and IMP2 with the C28 Zipcode RNA. From these data, I do not understand how the authors can derive a Kd of 161nM for the IMP2-C28 interaction since the interaction did not reach saturation at 2uM protein. I believe that the only conclusion here is that IMP2 has a lower affinity than ZBP1 for this RNA.

- ***We thank the reviewer for pointing this out. The gels shown in Fig 1c were chosen to make clear the difference in affinities for IMP2 and ZBP1 and this RNA. We apologize for the omission in the supplementary data of a more quantifiable gel for IMP2. To remedy this, we have now included the EMSAs where the IMP2 protein concentration was increased up to 8 uM (Supplementary Fig 5). We calculated the Kd of IMP2 for the C28 zipcode from this gel and it value is ~160 nM.***

2- It is not clear what is the RNA sequences described in Supplementary Figure 1 (RNA #1 and RNA #2).

- ***To make the sequences easier to read, we have now moved them to the supplementary table.***

Also in relation to Figure 1a, it would be useful to add a sequence alignment of the RNAs derived from SELEX round 9 as a supplementary figure.

- ***Due to the large number of RNA sequences from SELEX we have added a separate text file with all sequences processed from the SELEX (rounds 7, 8, 9) as source data.***

The authors should explain or speculate on the fact that 26% of the sequences contain a UAA motif at the 5'end instead of a UCA.

- ***Thank you for noticing this. We left out this in the discussion to avoid over interpretation of the SELEX RNAs. Our initial thought was that the 'CA' sequence requirement was less stringent than the 'GG'; this was also observed when point mutations of the CA region in the ß-actin zipcode did not dramatically affect binding to ZBP1/IMP1. This data is consistent with the observed differences in exchange rate when each motif is separately added into the NMR spectrum and the observed affinities of the individual fragments published from other laboratories (Nicastro et al, 2017, Cell Reports).***
- ***We also believe that this strengthens our findings that the U nucleotide is the more important for IMP2 recognition (Fig 5a) and its abundance in the SELEX may further support that idea.***

3- Most of the manuscript's conclusions are based on comparing the Kd of ZBP1 and IMP2 to different RNAs using quantifications of EMSA experiments (Figures 2c,d,e, and 5a,c,d, Sup Figs 1b,d, 2b). Are these derived from a

single EMSA experiment? There are no standard deviations and statistical analysis of the results. These should be added to demonstrate that the results presented are statistically significant.

- *We apologize for this omission. All quantifications were performed as previously published (Chao et al 2010, Genes and Development; Patel et al 2012, Genes and Development). The representative EMSAs and curves shown were from a single experiment but the Kd measurements were taken across multiple experiments, at least 3 independent experiments for each representative EMSA. We have now added these details into the reporting summary and modified the figure legends appropriately to show that the results presented are representative. Additionally, we have added standard errors in places where they were missing (Fig 4D).*

4- The figure presenting the NMR data (Fig. 3) is quite small and it is difficult to appreciate the changes in chemical shifts. Although Supp Figure 3 shows a larger version of the spectrum, it is not overlaid with the RNA bound form. Figure 3 A and B should be enlarged (possibly in a Supplementary Figure) and show an overlay of all the spectra (as in the examples shown in Supp. Fig. 3c and d). It would also be informative to add a bar chart of the chemical shift changes (or peak intensity change for KH4) as a function of the amino acid sequence.

- *We have now included enlarged spectra of our titrations as a supplementary figure panel (now Sup Fig. 3e) as well as both peak intensity changes and chemical shift changes (now Sup Fig. 3f).*

Also, what is the affinity of IMP2 to these RNA sequences?

- *The low uM affinity of the IMP2 SELEX fragments was approximated from the NMR titration (we extended the titration until the RNA was in excess of the protein). Our approximation is consistent with the previously published literature for ZBP1/IMP1 (1.5uM for KH3 and 0.9uM for KH4 from Nicastro et al 2017, Cell Reports). A single RNA containing both fragment sequences (the SELEX target) binds to IMP2KH34 in the low nM range due to increased avidity. This is explained in more detail below.*

It seems that the binding of KH3 to CCUCACC is in fast exchange and the binding of KH4 to UUUGGAAC is in intermediate exchange. This is not consistent with the described low nanomolar affinities obtained with EMSA.

- *We agree with the reviewer comment and apologize that this was not made clearer in the text. The EMSAs were done with full length RNA (ranging from 28-48nt). Unfortunately, the IMP2 KH34 complex with the longer RNAs is not soluble at concentrations required for NMR experiments.*
- *To gain an understanding of the binding sites, multiple approaches were taken. Extensive co-crystal trials were attempted but we were unable to achieve crystals of the RNA-protein complex.*
- *We then turned to dividing the RNA into two fragments, and individually titrating in each fragment into our NMR sample. This approach has been extensively used for RNA binding proteins (ZBP1, KSRP, NOVA etc). (Patel et al, 2012, Genes and Development; Nicastro et al, 2012, NSMB; Lewis et al, 2000, Cell)*

Finally, how does the NMR analysis of the affected residues correlates with previous NMR studies of ZBP1 KH3-KH4 with RNA?

- *The titration experiments of both ZBP1 (Patel et al 2012, Genes and Development) and IMP2 (this study) are consistent and show that GXXG and variable loop amino acids are perturbed in response to RNA.*

- *A separate laboratory developed two NMR based models of the RNA fragment bound complexes for ZBP1KH3 and ZBP1KH4 (Nicastro et al Cell Reports 2017) using two different ZBP1 mutants: "KH3-KH4DD (KH4 KO) protein bound to the target GCACACCC RNA and KH3DD-KH4 (KH3 KO) RNA bound to the UCGGACU RNA." NMR structures were solved for each of the two mutants. Then MD simulations were used to generate 100 different conformers of which the 12 lowest energy conformations were published as a part of the PDB.*
- *In these structures, the KH4 variable loop is near its RNA fragment. Our previous NMR titrations and the ITC data from the paper shows that this fragment is in intermediate exchange, has a higher affinity than KH3 and could participate in hydrogen bonding. The KH3 variable loop in contrast is displaced from the nucleobases of the RNA. Our previous NMR titrations and the ITC data from the paper shows that this fragment is in fast exchange, and has a lower affinity than KH4.*
- *There are a few possible reasons for this discrepancy – the most likely being that the use of RNA fragments in lieu of the full length zipcode sequence (for solubility reasons described above). As the variable loops are highly dynamic, it is possible that their position relative to the RNA changes in response to weak vs tight RNA binding. We would expect this change to be most apparent for the KH3 domain fragment (the CA motif) as it has a lower affinity for its target.*

5b. The mutations are based on a sequence alignment of ZBP1 and IMP2. However, the structures of ZBP1 KH3 and KH4 in complex with RNA have been solved and this manuscript describes NMR data that should provide a detailed description of the amino acids involved in the RNA binding (see point 4 above). Are the mutated residues involved in RNA binding either in the published structures, or in the NMR titration experiments?

- *Please find a detailed response above. A co-crystal of the full-length RNA bound to the KH34 domains could reconcile the discrepancies, however, this has not yet been possible and the success of crystallization cannot be predicted. Because of this we believe our functional data to be sufficient.*

5- The mutagenesis analysis done to provide a rationale for the differential RNA binding between ZBP1 and IMP2 is unclear to me:

5a. The previous experiments, especially SELEX, have shown that IMP2 binds a slightly different sequence than ZBP1. However, in the mutagenesis experiments, the authors have mutated IMP2 and investigated the binding of these mutants to the Zipcode RNA sequence. It would have been more logical to mutate ZBP1 residues and investigate their binding to the IMP2 RNA binding sequence obtained by SELEX.

- *We agree that this would have been a valid approach, however, we chose to consider the possible limitations of an artificially evolved SELEX RNA when deciding to investigate zipcode mutants.*
- *We believe the zipcode RNA to be a biologically relevant target as it has been extensively validated over the past few decades. In prior work, the SELEX for ZBP1 was used to support the binding to the zipcode by randomizing the zipcode sequence (Patel et al 2012, Genes and Development) but this was only possible because a well characterized RNA target containing the zipcode was available (i.e., ß-actin mRNA) .*
- *The cycle of characterizing the zipcode sequence occurred over the past 30 years and has involved multiple iterations of functional studies, mutagenesis and SELEX. This work represents a large leap forward for the characterization of IMP2. We believe further studies are absolutely needed to find biological targets and characterize the RNA-IMP2 protein relationship to the same extent of ß-actin zipcode-ZBP1 pair.*

- ***Because of this, we refrained from converting ZBP1 into an IMP2 SELEX binding protein but agree that when a canonical target of IMP2 is characterized, this experiment would be very interesting to perform.***

5c. Similarly, RNA mutations were done on the Zipcode RNA and therefore do not explain why the SELEX RNA sequence is preferentially bound by IMP2 and less so by ZBP1. It would have been more judicious to mutate the SELEX RNA and investigate the binding of ZBP1 to these mutants.

- ***As this is the first investigation of IMP2KH34's structure and consensus sequence we were hesitant to say that the SELEX RNA was a bona fide target and opted instead to mutate the well characterized zipcode.***

6- The genome wide search for IMP2 targets is highly speculative and only based on the identification of sequences that correspond to the SELEX sequence identified for IMP2. There is no experimental evidence that the identified sequences are real targets of IMP2. At least, the functional interaction of IMP2 with some of the mRNAs encoding for metabolic diseases such as Type II diabetes should be demonstrated experimentally.

- ***We agree and provide a new analysis of RIP data as we mentioned at the beginning of this letter (see details above). We observed that highly significant portion ($p < 0.0001$) of the predicted IMP2 consensus sequence targets were enriched in previously published RIP studies (Huang et al 2018, Nature Cell Biology; and Dai et al 2015, Cell Metabolism). We have now included these results in a new supplementary figure (Sup Fig. 7) and in the text, accordingly.***

Point-by-point response to the reviewers:

Reviewer #2 (Remarks to the Author):

The paper by Biswas and colleagues report on the structure and RNA binding properties of the KH3KH4 di-domain of the protein IMP2, one of three members of the IMP family in human. The authors show the structure of the di-domains of IMP2 is very similar to the one of the better characterized IMP1 paralogue. They then characterize the binding sequence of the di-domain and find that, while the two core positions of the sequence recognized by KH3 and KH4 are the same in IMP2 and IMP1, the bases flanking this di-nucleotide are different. They also show that these difference are due to sequence differences in the variable loop of the KH domains and that an IMP2 chimera with an IMP1 variable loop recognize the IMP1 cognate sequence. They search for targets containing this sequence and find that the identified putative IMP2 targets relate to metabolism control and that the overlap between putative targets containing the IMP1 and the IMP2 sequences could be significantly smaller than previously reported.

The paper is well-written and clear and the data present are logically organized and of good quality. It has a number of points of interest, including the structure of the di-domain, the novel sequence specificity, the creation of a chimera as a strategy to define specificity and, importantly, the definition of IMP1 and IMP2 sets of targets which have only a small overlap, which is different from what previously reported. Below are a number of comments to be addressed prior to publication.

1) The authors use the newly defined bi-partite RNA target motif to identify a number of bound 3'UTRs. They should directly validate the specificity of a selection these interactions. One possibility would be to use RIP or equivalent methods.

- ***We appreciate the reviewer's comments and provide a new analysis of RIP data, detailed at the beginning of this letter to validate the specificity of IMP2 consensus sequence. We observed that highly significant portion ($p < 0.0001$) of the predicted IMP2 consensus sequence targets were enriched in previously published RIP studies (Huang et al 2018, Nature Cell Biology; and Dai et al 2015, Cell Metabolism). We have now included these results in a new supplementary figure (Sup Fig. 7) and in the text, accordingly.***

2) The authors show that IMP2 sequence specificity is different than IMP1. The increase in affinity for IMP2, and decrease for IMP1, as we proceed in the SELEX selection for IMP2 is a good validation that strengthens their conclusions. However, my understanding is that in the discussion SELEX is implicitly equated to actual evolution - it should be clarified this is not the case.

- ***We agree that the evolution via SELEX may not reflex actual evolution. We have corrected the text accordingly to better separate the discussion of SELEX and evolution.***

3) The authors show that most of the targets that they attribute to IMP2 are linked to metabolic disorders. This I find very interesting and I think it would be worth discussing at the level of individual targets in the discussion.

- ***Thank you for this comment. The data is consistent with the published literature showing dramatic metabolic changes for mice or cells overexpressing or deficient in IMP2 (Janiszewska et al, 2012, Genes and Development; Dai et al, 2015, Cell Metabolism). It is also consistent with IMP2 being one of the***

few genes where SNPs are associated with diabetes (Scott et al, Science 2007; Zeggini et al, Science, 2007; Broad Institute and Novartis, Science, 2007).

- ***We have added a list of high confidence consensus sequence targets (conserved between mouse and human) that were also highly enriched in RIP studies (Huang et al 2018, Nature Cell Biology; and Dai et al 2015, Cell Metabolism) and related to metabolism and diabetes.***
- ***Interestingly, RNA encoding the anti-diabetic protein GRB10 was enriched in both data sets. We were surprised to learn that GRB10 overexpression phenocopies the IMP2KO and have added a small discussion point hypothesizing that dysregulation of IMP2 and GRB10 may be related to diabetes.***

4) The overlap between the targets of IMP1 and IMP2 is much smaller than the one observed in CLIP studies. This is important, as it would change significantly the way we think about the role and targets of these proteins. It should be discussed more in my opinion.

- ***Investigating the discrepancies between in vitro RNA-protein specificity and the broad range of RNA targets in vivo is very challenging and require further studies. We agree that this difference in overlap is very interesting. This is now further discussed in the text.***

5) Changing the sequence specificity of a KH domain by the mutation of an individual amino acid has been previously shown to be possible by Nicastro et al., NSMB 2012. This should be mentioned appropriately when discussing mutational strategies in more general terms.

- ***We thank the reviewer for this reference and have now included it in our discussion. We noticed that the K368R mutation described in Nicastro et al., NSMB 2012, is in the KH3 variable loop of KSRP and reduced affinity to the target RNA by three to four fold. However, we found a combination of amino acid mutations that increase the affinity of binding. To the best of our knowledge our work represents the first gain of function for KH domain containing proteins.***

6) The authors test the relevance of the variable loop in IMP2. What they find may not be true for other KH domains, for example the ones showing very little sequence specificity in 'position 1' of the bound sequence. This should be considered in the discussion.

- ***We agree with the reviewer comment and have now modified our statements in the discussion.***

REVIEWERS' COMMENTS:

Reviewer #1 (Remarks to the Author):

The authors have answered most of my concerns and modified the manuscript accordingly. I have no further concerns

Reviewer #2 (Remarks to the Author):

In the revised version of the manuscript of Biswas et al. the authors have met most of my concerns. There are still a couple of minor points in the text that need to be addressed prior to publication.
1) Biswas and co-workers identify a number of putative targets based on the novel consensus sequence. In the revised version, show these targets are part of an ensemble of IMP2 targets that has been identified experimentally using RIPseq. The strong correlation they observe largely addresses my concerns. However, I have two of comments on this: i) in page 11 line 2 the authors define the RNA that contain the KH2 binding motif conserved in human and mice as high confidence targets. As I understanding it, at this point these are putative targets, the authors have no experimental evidence, from them or others, to show that these are actual targets. I am not sure about using 'high confidence targets' at this stage ii) More important, my understanding is that the overlap that exists between the IMP1 and IMP2 targets from the published the RIPseq/PARCLIP data from Huan et al. is extensive, while the one observed in the targets identified by Biswas et al. using the IMP2 sequence motif is very small, despite the IMP2 targets been highly correlated. Could the authors comments briefly on this?
2) Page 13, lines 3 and 4: the authors state that this study addresses the search of randomized linker regions between specific binding motifs. Work by Patel et al., in G&D 2012 and more recently by Schneider et al 2019 in Nat. Comm also explores this problem. The current work should be placed in the context of those earlier papers here.
3) Page 13, the authors state that 'This study provides the first gain of function mutation'… . I would use 'Gain of RNA-binding function' here. A generic 'Gain of function' statement is typically used in studies where the function is directly tested in vivo, and this is not done here.

REVIEWERS' COMMENTS:

Reviewer #1 (Remarks to the Author):

The authors have answered most of my concerns and modified the manuscript accordingly. I have no further concerns

- ***Thank you for your comments and suggestions – they helped to improve the manuscript significantly.***

Reviewer #2 (Remarks to the Author):

In the revised version of the manuscript of Biswas et al. the authors have met most of my concerns. There are still a couple of minor points in the text that need to be addressed prior to publication.

1) Biswas and co-workers identify a number of putative targets based on the novel consensus sequence. In the revised version, show these targets are part of an ensemble of IMP2 targets that has been identified experimentally using RIPseq. The strong correlation they observe largely addresses my concerns. However, I have two of comments on this:

i) in page 11 line 2 the authors define the RNA that contain the KH2 binding motif conserved in human and mice as high confidence targets. As I understanding it, at this point these are putative targets, the authors have no experimental evidence, from them or others, to show that these are actual targets. I am not sure about using 'high confidence targets' at this stage

- ***We agree with the reviewer comment that the choice of 'high confidence' may be ambiguous, although we correlated the presence of the consensus sequence with RIPseq and functional annotations. We have now replaced 'high confidence' targets with 'evolutionarily conserved' to emphasize that these targets have had the IMP2 consensus sequence conserved in their 3' UTRs from mouse to human.***

ii) More important, my understanding is that the overlap that exists between the IMP1 and IMP2 targets from the published the RIPseq/PARCLIP data from Huan et al. is extensive, while the one observed in the targets identified by Biswas et al. using the IMP2 sequence motif is very small, despite the IMP2 targets been highly correlated. Could the authors comments briefly on this?

- ***We agree that this is an important point and may reflect differences between the in vitro studies focus on the KH34 domains (for both ZBP1 and IMP2) and the use of full length protein for RIPseq/PARCLIP approaches. We have highlighted this comment as stated in the text:***

  "Modification of binding preferences by additional domains may explain some of the discrepancies between highly specific in vitro binding and broad binding highly overlapping targets in vivo. This metabolic role for IMP2 as well as its divergence from ZBP1 was clear from mouse studies, however high throughput studies often find significant overlap in the target pools. Reconciling the specificity differences of these proteins when comparing top down vs bottom up approaches remains a challenge and could benefit from orthogonal techniques to validate in vivo binding."

2) Page 13, lines 3 and 4: the authors state that this study addresses the search of randomized linker regions between specific binding motifs. Work by Patel et al., in G&D 2012 and more recently by Schneider et al 2019 in Nat. Comm also explores this problem. The current work should be placed in the context of those earlier papers here.

- ***We thank the reviewer for this comment. We have now modified the text accordingly to show our work in the context of earlier papers:***

  "Alongside previous work (Patel et al, 2012; Schneider et al, 2019), this study incorporates randomized variable linker regions between different domain consensus sequences when performing in silico target searches. The increased flexibility between consensus sequence elements helps discover targets of the IMP family and may inform future genome wide searches of other RBP targets."

3) Page 13, the authors state that 'This study provides the first gain of function mutation'… . I would use 'Gain of RNA-binding function' here. A generic 'Gain of function' statement is typically used in studies where the function is directly tested in vivo, and this is not done here.

- ***Thank you for this comment. The text has been changed accordingly***
